# The clinical significance and oncogenic function of LRRFIP1 in pancreatic cancer

**DOI:** 10.1007/s12672-024-00977-3

**Published:** 2024-04-18

**Authors:** Jinping Li, Dayun Tuo, Gunan Guo, Yan Gao, Jinfeng Gan

**Affiliations:** 1 Department of Histology and Embryology, School of Preclinical Medicine, Guilin Medical University, Guilin, 541199 Guangxi People’s Republic of China; 2 Department of Pathology, Liuzhou People’s Hospital, Liuzhou, 545006 Guangxi People’s Republic of China; 3 School of Stomatology, Zhaoqing Medical College, Zhaoqing, 526020 Guangdong People’s Republic of China; 4 Guangxi Key Laboratory of Tumor Immunology and Microenvironmental Regulation, Guilin Medical University, Guilin, 541199 Guangxi People’s Republic of China; 5 Department of Gastroenterology, The Second Affiliated Hospital of Guilin Medical University, Guilin, 541199 Guangxi People’s Republic of China

**Keywords:** Pancreatic cancer, Prognosis, LRRFIP1, AKT, GSK-3β, β-catenin

## Abstract

**Purpose:**

Pancreatic cancer is a lethal malignancy with a grim prognosis. Previous studies have proven that Leucine Rich Repeat of Flightless-1 Interacting Protein 1 (LRRFIP1) plays a pivotal role in cell biological processes, while its clinical significance and function in pancreatic cancer remain to be elucidated. Hence, we aimed to explore the roles and mechanisms of LRRFIP1 in pancreatic cancer.

**Methods:**

The expression of LRRFIP1 in pancreatic cancer tissues and its clinical significance for pancreatic cancer were analyzed by immunohistochemistry assay and bioinformatic analysis. The influences of LRRFIP1 on the proliferation and migration of pancreatic cancer cells were assessed in vitro. The underlying mechanisms of LRRFIP1 in pancreatic cancer progression were explored using gene set enrichment analysis (GSEA) and molecular experiments.

**Results:**

The results showed that LRRFIP1 expression was significantly upregulated in pancreatic cancer tissues compared to the normal tissues, and such upregulation was associated with poor prognosis of patients with pancreatic cancer. GSEA revealed that LRRFIP1 upregulation was significantly associated with various cancer-associated signaling pathways, including PI3K/AKT signaling pathway and Wnt pathway. Furthermore, LRRFIP1 was found to be associated with the infiltration of various immune cells. Functionally, LRRFIP1 silencing suppressed cell proliferation somewhat and inhibited migration substantially. Further molecular experiments indicated that LRRFIP1 silencing inactivated the AKT/GSK-3β/β-catenin signaling axis.

**Conclusion:**

Taken together, LRRFIP1 is associated with tumorigenesis, immune cell infiltration, and prognosis in pancreatic cancer, which suggests that LRRFIP1 may be a potential biomarker and therapeutic target for pancreatic cancer.

**Supplementary Information:**

The online version contains supplementary material available at 10.1007/s12672-024-00977-3.

## Introduction

Pancreatic cancer is one of the most lethal cancers in the world, with a 5-year survival rate of approximately 10% [1]. Surgical intervention is a top priority in the treatment of pancreatic cancer, but most of the patients are diagnosed at an advanced stage, missing out on surgery. In recent years, immunotherapy, such as immune checkpoint blockade, has achieved remarkable efficacy in many malignancies [2], but it remains a major challenge due to the lack of specific biomarkers for pancreatic cancer. Currently, carcinoembryonic antigen 19–9 (CA19-9) is the most commonly used tumor-associated antigen for the serological diagnosis and prognosis evaluation of pancreatic cancer [3], but its specificity in distinguishing pancreatic cancer from biliary diseases is not satisfactory. Therefore, it is imperative to identify some novel biomarkers and therapeutic targets for improving the diagnosis and prognosis of pancreatic cancer.

The Leucine Rich Repeat of Flightless-1 Interacting Protein 1/GC-binding factor 2 (LRRFIP1/GCF2) gene contains eight exons and introns and encodes 752 amino acid residues with a detected molecular mass of 160 kDa. The molecular structure of the LRRFIP1 protein consists of three domains: a helix domain, a central coiled-coil domain, and a nucleic acid binding domain, which is correlated with its functions such as transcriptional inhibition, signal transduction, and cytoskeleton rearrangement [4]. Some studies have revealed that LRRFIP1 is an oncogene that is highly expressed in multiple malignant tumors [4–7]. Moreover, some researchers have confirmed that high LRRFIP1 expression is associated the cancer progression, including cancer cell proliferation, invasion, metastasis, and drug resistance [7–11]. In pancreatic cancer cells, silencing of LRRFIP1 can reverse the epithelial-mesenchymal transition (EMT) [12], and increase gemcitabine sensitivity [13]. Therefore, LRRFIP1 plays a critical role in pancreatic cancer. However, the clinical significance and function of LRRFIP1 in pancreatic cancer are yet to be defined.

In the present study, we evaluated LRRFIP1 expression in pancreatic cancer tissues and investigated its clinical significance. Furthermore, bioinformatics analysis was used to investigate the signaling pathways that LRRFIP1 may be involved in, as well as its possible role in immune cell infiltration. Lastly, in vitro studies were performed to explore its biological functions and underlying mechanisms in pancreatic cancer progression.

## Materials and methods

### Human pancreatic cancer specimens and immunohistochemistry assay

A tissue microarray containing pancreatic cancer (n = 10) and paired adjacent normal tissues was purchased from Shanghai Outdo Biotech Company (Shanghai, China). The tissue microarray was processed and incubated with an anti-LRRFIP1 antibody (SANTA CRUZ, #SC-101168), followed by secondary antibody incubation. 3, 3'-diaminobenzidine (DAB) was used to detect the signal, and hematoxylin was used to counterstain the nuclei. The intensity of the staining was graded as 0 (negative), 1 (weak), 2 (moderate), and 3 (strong). The percentage of positively stained cells was divided into four categories: 0 (negative), 1 (1–25% positive cells), 2 (26–50% positive cells), 3 (51–75% positive cells), and 4 (> 75% positive cells) [14]. The immunohistochemistry score was calculated by multiplying the staining intensity by the percentage of positive tumor cells.

### TCGA and GEO datasets analyses

Transcriptional expression data of pancreatic cancer and corresponding clinical data were downloaded from The Cancer Genome Atlas (TCGA) portal (https://portal.gdc.cancer.gov/), which contains 179 pancreatic cancer samples and 4 paracancerous tissues. Meanwhile, 167 normal samples were obtained from Genotype-Tissue Expression (GTEx) database (https://gtexportal.org/). For further analysis, RNA-Seq data with a workflow type of Fragments Per Kilobase per Million (FPKM) was transformed into Transcripts per million reads (TPM) format, followed by log_2_ conversion [15]. Gene Expression Omnibus (GEO) is a public comprehensive gene expression library in the National Center of Biotechnology Information (https://www.ncbi.nlm.nih.gov/geo/) [16]. The GEO dataset GSE16515 [17] was chosen to analyze LRRFIP1 mRNA expression in pancreatic cancer and normal tissues.

### Clinical proteomic tumor analysis consortium (CPTAC) analysis

CPTAC (http://ualcan.path.uab.edu/analysis-prot.html) is a portal for cancer proteomics research. In the present study, we used CPTAC to investigate the level of LRRFIP1 protein expression in pancreatic cancer.

### The human protein Atlas (HPA) analysis

HPA (http://www.proteinatlas.org/) is a protein expression database based on immunohistochemical analysis that contains information about the protein expression profiles of most human genes in tumor and normal tissues. In this study, we used HPA to investigate the protein expression of LRRFIP1 in pancreatic cancer and normal pancreas tissues.

### Survival analysis

The TCGA pancreatic cancer patients were divided into two groups, namely, high- and low-expression groups, based on the expression value of LRRFIP1 (cutoff: upper 50% vs. lower 50%). The "Survival" package (version 3.6) in the R package was used to analyze the association between LRRFIP1 and overall survival in pancreatic cancer patients. The "survminer" package was used for visuals.

### Enrichment analysis

To investigate the potential biological function of LRRFIP1 in pancreatic cancer, we selected the top 210 genes that were most positively correlated with LRRFIP1 in TCGA pancreatic cancer for enrichment analysis. The EnrichGO function of the R package "clusterProfiler" was used to perform gene ontology (GO) enrichment, including biological process (BP), cell component (CC), molecular function (MF), and Kyoto encyclopedia of genes and genomes (KEGG) [18]. In addition, the EnrichKEGG function in the R package "clusterProfiler" was used to perform KEGG analysis.

### Construction of protein–protein interaction (PPI) networks

STRING (https://www.string-db.org/) is an online database for analyzing protein–protein interaction networks. In this study, we utilized STRING to search the proteins that interacted with LRRFIP1 (interaction score > 0.70) and construct a PPI network. We also analyzed the correlation between LRRFIP1 and the top ten possible interacting proteins according to the PPI network.

### Gene set enrichment analysis (GSEA)

GSEA was performed with the R package "clusterProfiler" to analyze the differences in function and pathway terms between the high-LRRFIP1 and low-LRRFIP1 expression groups. A function or pathway term with a *p*-value < 0.05 was considered to be a statistically significant enrichment.

### Immune cell infiltration analysis

To evaluate the role of LRRFIP1 expression in immune infiltration in pancreatic cancer, we investigated the association between LRRFIP1 expression and 24 different types of immune cells using the single-sample GSEA (ssGSEA) method in the R package GSVA (Version 1.34.0) [19, 20]. Moreover, the infiltration levels of immune cells in high- and low-LRRFIP1 expression groups were compared.

### Cell culture and small interference RNA (siRNA) transfection

Human pancreatic cancer cell lines MIAPaCa-2 and PANC-1 were cultured in Dulbecco's modified Eagle's medium with 10% fetal bovine serum (Gibco, USA), 100 U/ml penicillin, and 100 mg/ml streptomycin at 37 °C with 5% CO_2_.

The siRNA targeting LRRFIP1 (siLRRFIP1) and scrambled siRNA control (siNC) were purchased from GenePharma Co., Ltd (Shanghai, China). The siLRRFIP1 sequence was based on a previous study [13], and was as follows: sense: 5'-GGAAAUCAAGGACUCUCUAGCAGAA-3'. The siNC sequence was as follows: sense: 5'-UUCUUCGAACGUGUCACGUTT-3'. Pancreatic cancer cells were seeded into a 6-well plate. After reaching 70% confluency, the cells were transfected with siLRRFIP1 (100 pmol) and siNC (100 pmol) using Lipofectamine 3000 reagent (Invitrogen, USA). After 48 h, subsequent experiments were carried out on the transfected cells.

### Quantitative real-time polymerase chain reaction (qRT-PCR) assay

Total RNA was isolated from cells using Trizol reagent (Tiangen Biotech, Beijing, China) according to the manufacturer's instructions. Extracted RNA was reverse-transcribed using PrimeScript RT-polymerase (Takara, Beijing, China) following the manufacturer's instructions. qRT-PCR was performed using specific primers (Table S1) for target genes. GAPDH was used as an internal control and the 2^–∆∆Ct^ method was used for comparative quantitative analysis [21].

### Cell viability assay

Cells that transfected with siLRRFIP1 or siNC were seeded in a 96-well plate and cell viability was then determined using the Cell Counting Kit-8 (CCK-8) (APExBIO, #K1018) at 0, 24, 48, and 72 h. A multifunctional microplate reader (Tecan Group Ltd, Switzerland) was used to measure the absorbance at 450 nm (OD450).

### Cell migration assays

For wound healing assay, cells were seeded into a 6-well plate and transfected with siLRRFIP1 or siNC. When the cells achieved 90% confluence, they were scratched with a 200-μL pipette tip and subsequently cultured in the serum-free medium. After 24 h, images of the wound healing were taken and quantified using ImageJ software. The wound healing rate was calculated as follows: wound healing rate = (initial area of the wound–the wound area at 24 h)/initial area of the wound × 100%. In addition, transwell cell assay was performed in the transwell system with an 8-μm pore membrane (JET BIOFIL, China). Briefly, the transfected cells (1.5 × 10^5^ cells/well) were seeded in the upper chamber with serum-free culture medium, while the lower chamber contained medium with 20% fetal bovine serum. At 48 h, the migrated cells were fixed with 4% paraformaldehyde for 15 min, followed by staining with 0.1% crystal violet for 10 min at room temperature, and then images were taken with an Olympus CKX53 inverted microscope. The number of migrated cells was quantified by ImageJ.

### Western blotting assay

Cells were harvested and lysed in RIPA buffer (Beyotime, #P0013E) at 48 h after siRNA transfection. Protein concentration was detected using a BCA kit (Beyotime, #P0012S). The extracted protein was separated in a 10% sodium dodecyl sulfate–polyacrylamide gel electrophoresis (SDS-PAGE) and then transferred to nitrocellulose membranes. The membranes were blocked in TBST (Tris-buffered saline, 0.1% Tween 20) buffer containing 5% silk milk powder for 2 h at room temperature, then cut according to the molecular weight of the target proteins, and incubated with primary antibodies at 4 °C overnight. The membranes were washed three times followed by incubation with secondary antibody for 1 h at room temperature. The ECL system and Image Lab software were used to detect signals and analyze protein quantitatively, respectively. The primary antibodies used were as follows: anti-LRRFIP1 (Santa Cruz Biotechnology, #sc-101168, 1:500 dilution), anti-p-AKT (Cell Signaling Technology, #4060, 1:1000 dilution), AKT (Cell Signaling Technology, #4691, 1:1000 dilution), anti-p-GSK-3β (Beyotime, #AG753, 1:1000 dilution), anti-GSK-3β (Beyotime, #AG751, 1:1000 dilution), anti-β-catenin (Beyotime, #AF0069, 1:2000 dilution), and anti-GAPDH (ZS-GB Biotech, #TA-08, 1:5000 dilution).

### Statistical analysis

R (V 3.6.3) was used for all statistical analyses. The differences between two sets of data were compared using Wilcoxon rank sum test or Student's t-test, where appropriate. A value of *p* < 0.05 was considered as a statistical difference.

## Results

### LRRFIP1 is highly expressed in pancreatic cancer tissues

**Fig. 1:**
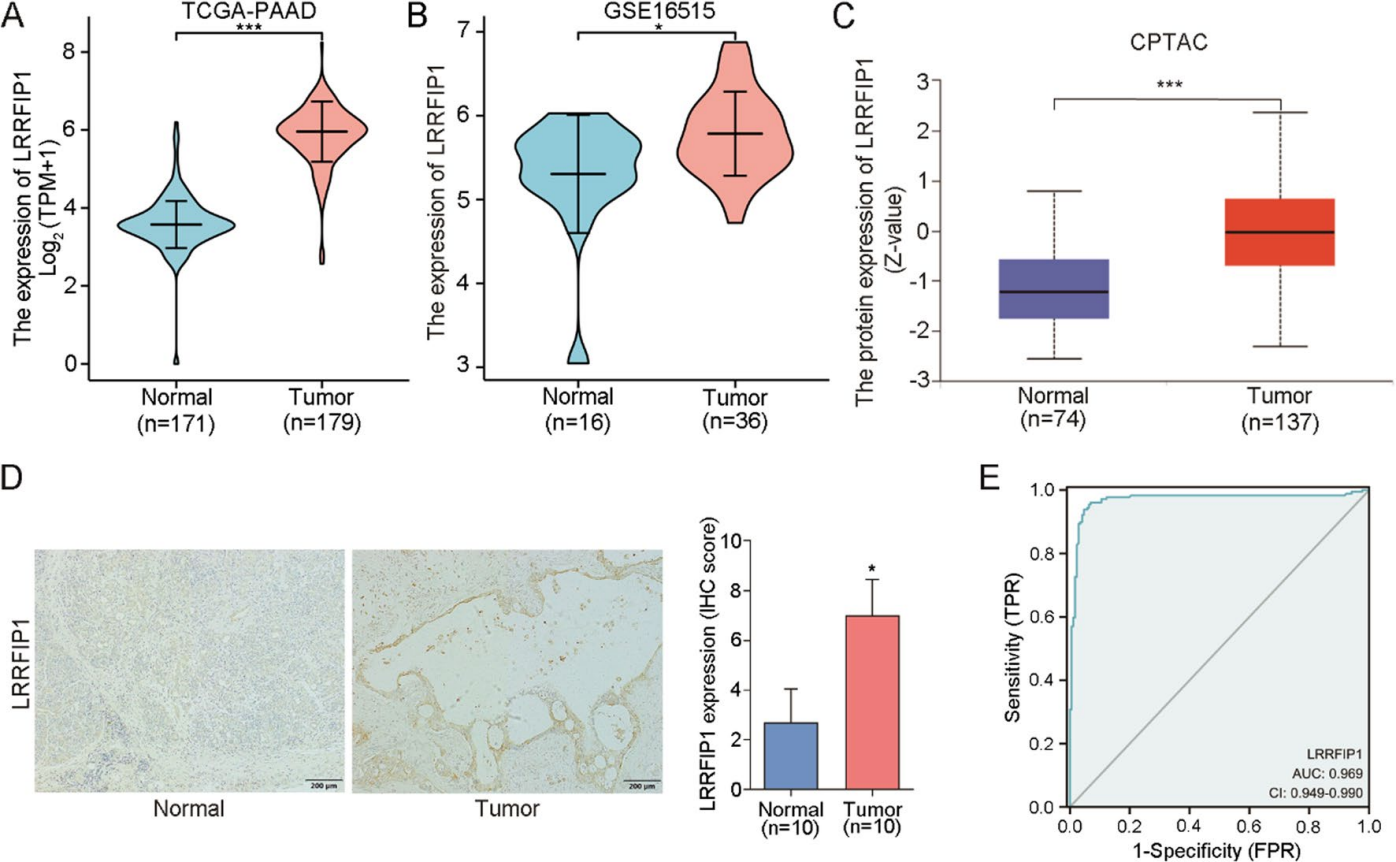
LRRFIP1 is highly expressed in pancreatic cancer tissues. **A**, **B** The mRNA expression level of LRRFIP1 in pancreatic cancer tissues and normal tissues was investigated in TCGA cohort (**A**) and GEO pancreatic cancer dataset GSE16515 (**B**). **C** The protein expression level of LRRFIP1 in pancreatic cancer tissues and normal tissues was analyzed in the CPTAC database. **D** Immunohistochemistry (IHC) analysis of LRRFIP1 in pancreatic cancer tissues and normal tissues (left), and the IHC staining was quantified (right). **E** ROC curve was applied to assess the accuracy of LRRFIP1 for discriminating TCGA pancreatic cancer tissues from normal tissues. ^*^p < 0.05, ^***^p < 0.001 by Wilcoxon rank sum test (**A**, **B**) or Student's t-test (**C**, **D**)

To investigate LRRFIP1 mRNA and protein expression in pancreatic cancer, we extracted LRRFIP1 expression data from TCGA, GEO, CPTAC, and HPA. The results showed that compared to normal tissues, LRRFIP1 mRNA expression was significantly upregulated in pancreatic cancer tissues (Fig. 1A, B , p< 0.05). Similar results were obtained on the protein expression level of LRRFIP1 (Fig. 1C, D, and Fig. S1). Given that LRRFIP1 expression was upregulated in pancreatic cancer tissues, we used receiver operating characteristic (ROC) curve analysis to assess LRRFIP1's diagnostic value for pancreatic cancer. The result showed that LRRFIP1 had an area under the curve (AUC) of 0.969 (confidence interval (CI): 0.949–0.990) (Fig. 1E) in discriminating pancreatic cancer tissues from normal tissues. Collectively, these results suggest that the expression of LRRFIP1 is upregulated in pancreatic cancer and LRRFIP1 has the potential to distinguish pancreatic cancer patients from healthy subjects.

### Upregulated LRRFIP1 expression predicts poor prognosis in pancreatic cancer patients

**Fig. 2:**
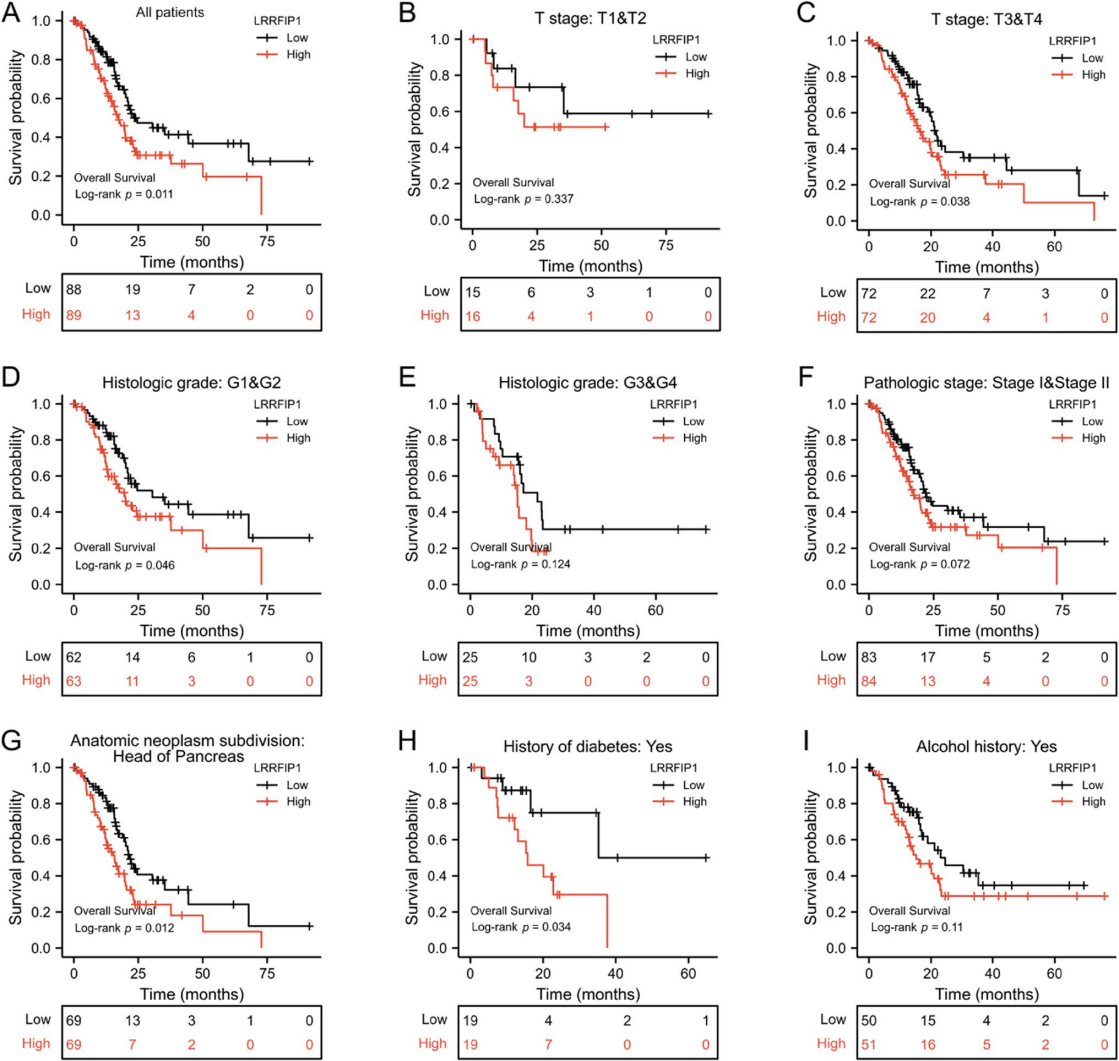
Correlation between LRRFIP1 and prognosis in pancreatic cancer patients. **A** High LRRFIP1 expression indicates shorter overall survival in all pancreatic cancer patients. **B**–**I** Association of LRRFIP1 and overall survival in pancreatic cancer patients in several subgroups, including T stage: T1&T2 (**B**), T3&T4 (**C**); histologic grade: G1&G2 (**D**), G3&G4 (**E**); pathologic stage: Stage I&Stage II (**F**); anatomic neoplasm subdivision: head of pancreas (**G**); history of diabetes (**H**) and alcohol history (**I**)

To better understand the relationship between LRRFIP1 expression and overall survival in pancreatic cancer patients from TCGA, a Kaplan–Meier survival analysis was performed. As shown in Fig. 2A, the patients with high LRRFIP1 expression had a shorter overall survival than those patients with low LRRFIP1 expression (Fig. 2A, log-rank *p* = 0.011). Furthermore, the subgroup analyses revealed a significant relationship between high LRRFIP1 expression and overall survival in pancreatic cancer patients with various clinical features, including patients with T3&T4 (Fig. 2C, log-rank *p* = 0.038), G1&G2 (Fig. 2D, log-rank *p* = 0.046), head of pancreas (Fig. 2G, log-rank *p* = 0.012), and history of diabetes (Fig. 2H, log-rank *p* = 0.034). However, in the patients with T1&T2 (Fig. 2B, log-rank *p* = 0.337), G3&G4 (Fig. 2E, log-rank *p* = 0.124), Stage I &Stage II (Fig. 2F, log-rank *p* = 0.072), or alcohol history (Fig. 2I, log-rank *p* = 0.11), elevated LRRFIP1 expression did not significantly relate with overall survival. Overall, the data suggest that upregulated expression of LRRFIP1 in pancreatic cancer may lead to poor prognosis.

To further investigate the clinical significance of LRRFIP1 in pancreatic cancer, we also analyzed the relationship between LRRFIP1 expression, clinical characteristics, and prognosis of pancreatic cancer patients by univariate and multivariate Cox regression analysis, respectively. As shown in Table 1, clinical T stage (Hazard ratio (HR) = 2.040, 95% CI 1.082–3.850, *p* = 0.028), N stage (HR = 2.106, 95% CI 1.254–3.539, *p* = 0.005), residual tumor (HR = 1.625, 95% CI 1.044–2.530, *p* = 0.032), and anatomic neoplasm subdivision (HR = 2.332, 95% CI 1.291–4.213, *p* = 0.005), radiation therapy (HR = 0.502, 95% CI: 0.295–0.856, *p* = 0.011), primary therapy outcome (HR = 0.371, 95% CI 0.228–0.603, *p* < 0.001), and LRRFIP1 (HR = 1.716, 95% CI 1.129–2.608, *p* = 0.012) were significantly correlated with the overall survival of pancreatic cancer patients. In addition, histologic grade was found to be marginally correlated with overall survival in pancreatic cancer patients (HR = 1.523, 95% CI 0.986–2.352, *p* = 0.058). The above factors were further subjected to multivariate Cox regression analysis and the results revealed that histologic grade (HR = 1.835, 95% CI 1.062–3.169, *p* = 0.029), radiation therapy (HR = 0.423, 95% CI 0.220–0.816, P = 0.010), and primary therapy outcome (HR = 0.491, 95% CI 0.275–0.876, *p* = 0.016) were significantly correlated with pancreatic cancer patient's prognosis, but LRRFIP1 failed to be an independent prognostic factor for overall survival of pancreatic cancer patients among the factors examined (HR = 1.287, 95% CI 0.781–2.119, *p* = 0.322). These results suggest that LRRFIP1 is related to the prognosis of pancreatic cancer patients.

**Table 1:** Univariate and multivariate analysis of the prognostic value of clinicopathological characteristics in pancreatic cancer patients' overall survival

Characteristic	Univariate analysis		Multivariate analysis	
	HR (95% CI)	*p*-value	HR (95% CI)	*p*-value
Age (> 65 vs. < = 65)	1.311 (0.868–1.980)	0.198		
Gender (Male vs. Female)	0.820 (0.544–1.235)	0.343		
T stage (T3&T4 vs. T1&T2)	2.040 (1.082–3.850)	0.028	1.024 (0.488–2.149)	0.950
N stage (N1 vs. N0)	2.106 (1.254–3.539)	0.005	1.530 (0.796–2.940)	0.202
Pathologic stage (Stage III &Stage IV vs. Stage I &Stage II)	0.800 (0.252–2.537)	0.704		
Histologic grade (G3&G4 vs. G1&G2)	1.523 (0.986–2.352)	0.058	1.835 (1.062–3.169)	0.029
Residual tumor (R1&R2 vs. R0)	1.625 (1.044–2.530)	0.032	1.468 (0.834–2.583)	0.184
Anatomic neoplasm subdivision( Head of Pancreas vs. Other)	2.332 (1.291–4.213)	0.005	2.071 (0.963–4.454)	0.063
Radiation therapy (Yes vs. No)	0.502 (0.295–0.856)	0.011	0.423 (0.220–0.816)	0.010
Primary therapy outcome (CR vs. PD&SD&PR)	0.371 (0.228–0.603)	< 0.001	0.491 (0.275–0.876)	0.016
Alcohol history (Yes vs. No)	1.125 (0.724–1.749)	0.601		
History of chronic pancreatitis (Yes vs. No)	1.177 (0.562–2.464)	0.666		
History of diabetes (Yes vs. No)	0.927 (0.532–1.615)	0.790		
LRRFIP1 (High vs. Low)	1.716 (1.129–2.608)	0.012	1.287 (0.781–2.119)	0.322

*HR* hazard ratio; *CI* confidence interval; *CR* complete response; *PD* progressive disease; *SD* stable disease; *PR* partial response

### The LRRFIP1-correlated genes are enriched in various biological terms in pancreatic cancer

**Fig. 3:**
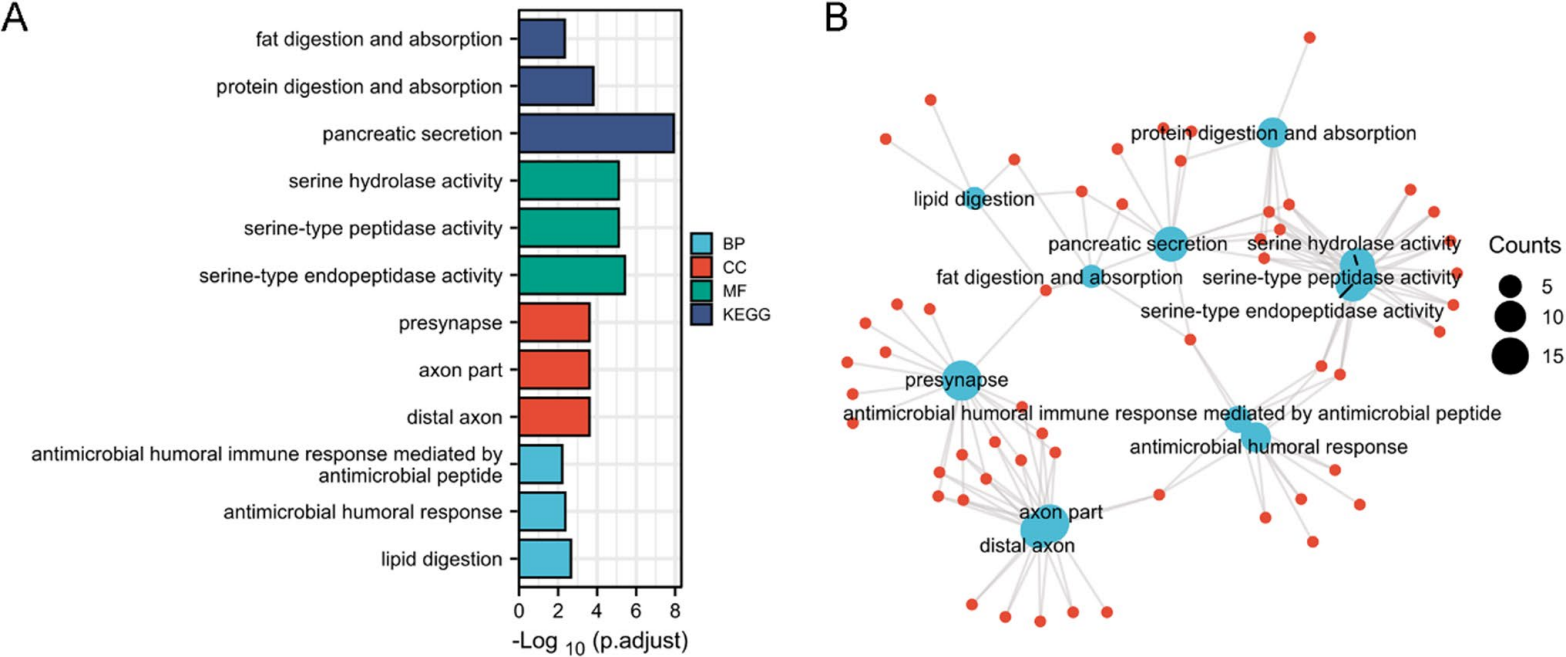
Function and pathway enrichment analysis of LRRFIP1 in pancreatic cancer. **A** Significant GO terms (including BP, MF, CC, and KEGG) of the top 210 genes most positively associated with LRRFIP1. **B** Significant KEGG pathway of the top 210 genes most positively associated with LRRFIP1

**Fig. 4:**
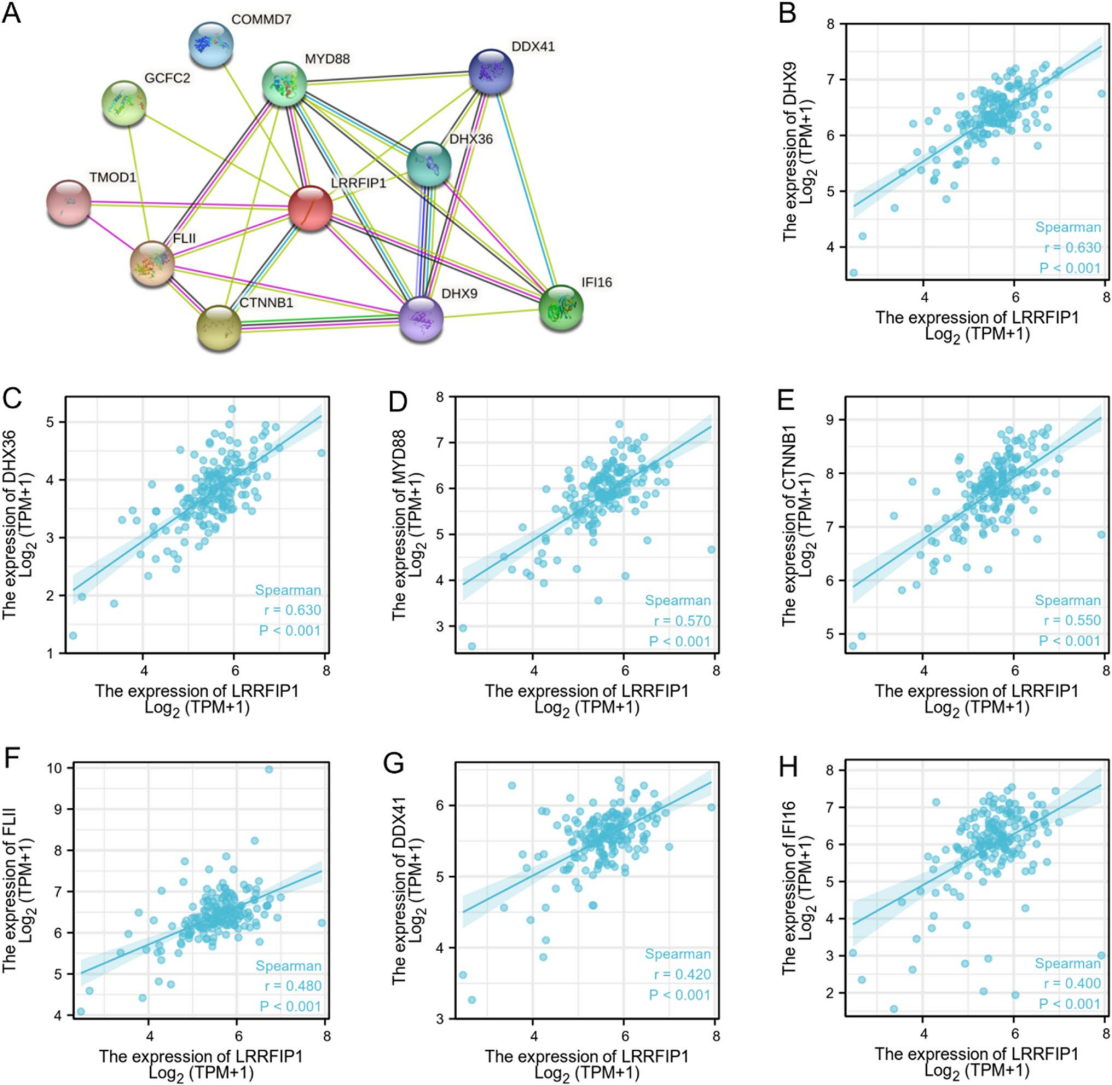
Construction of LRRFIP1-related PPI networks. **A** A network of LRRFIP1-possible interacting proteins was generated by the STRING database. **B–H** Correlation analysis of LRRFIP1 and DHX9 (**B**), DHX36 (**C**), MYD88 (**D**), CTNNB1 (**E**), FLII (**F**), DDX41 (**G**), IFI16 (**H**). DHX9, ATP-dependent RNA helicase DHX9; DHX36, ATP-dependent RNA helicase DHX36; MYD88, Myeloid differentiation primary response protein 88; CTNNB1, Catenin beta-1; FLII, Protein flightless-1 homolog; DDX41, Probable ATP-dependent RNA helicase DDX41; IFI16, Gamma-interferon-inducible protein 16

To investigate the potential functions of LRRFIP1 in pancreatic cancer, we searched the TCGA database for LRRFIP1 expression-correlated genes in the pancreatic cancer cohort to analyze the involved pathways. We identified the top 210 genes that most positively correlated with LRRFIP1, and these genes were subjected to gene ontology (GO) and KEGG enrichment analysis using the "clusterProfile" R package. The GO analysis showed that the majority of the genes were associated with multiple molecular functions, including "serine hydrolase activity", "serine-type peptidase activity", and "serine-type endopeptidase activity" (Fig. 3A). Meanwhile, the KEGG data revealed that LRRFIP1 may be related to "pancreatic secretion", "protein digestion and absorption", and "fat digestion and absorption" (Fig. 3B). We further used the STRING database to build a PPI network of LRRFIP1 and the top ten potential interacting proteins were shown in Fig. 4A. The correlation analyses between LRRFIP1 expression and some potential interacting proteins (r ≥ 0.4 and *p* < 0.001) were shown in Fig. 4B–H. We further verified the effects of LRRFIP1 on the expression of these genes. Consistent with bioinformatics predictions, qRT-PCR assay showed that LRRFIP1-knockdown significantly downregulated mRNA expression of most of the genes in MIAPaCa-2 cells (Fig. S2). Altogether, the above data demonstrate that LRRFIP1 may play an important role in pancreatic cancer by regulating various signaling pathways.

### LRRFP1 is correlated with multiple signaling pathways in pancreatic cancer

**Fig. 5:**
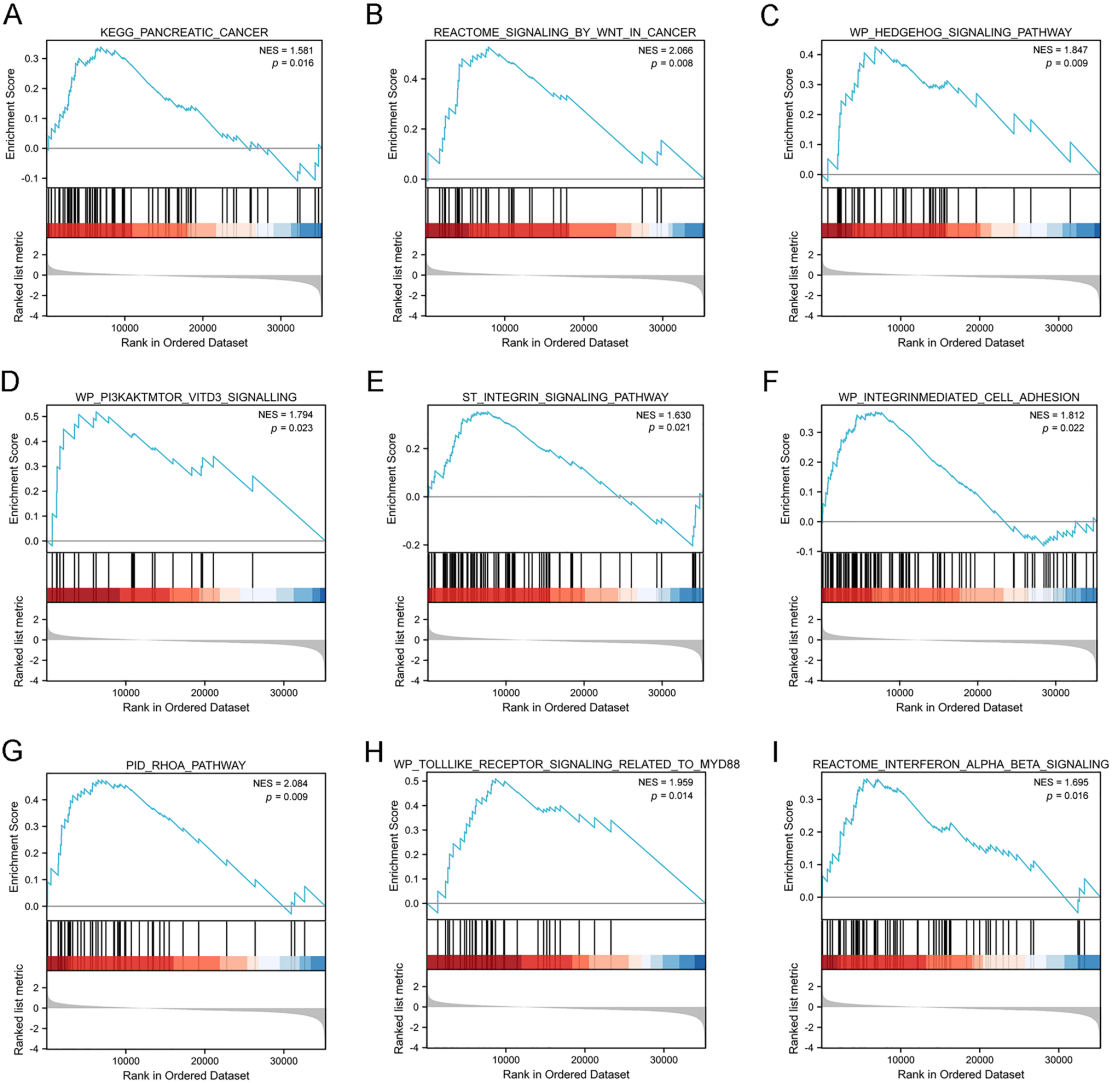
LRRFIP1 is correlated with multiple pathways in pancreatic cancer. GSEA analysis was used to identify signaling pathways associated with LRRFIP1. Nine pathways, including "Pancreatic cancer signalling" (**A**), "Signaling by Wnt in cancer" (**B**),"Hedgehog signaling pathway" (**C**), "PI3K/AKT/mTOR_VITD3 signaling" (**D**), "Integrin signaling pathway" (**E**), "Integrin-mediated cell adhesion signalling" (**F**), "RhoA pathway" (**G**), "Toll-like receptor signaling related to MYD88" (**H**), and "Interferon alpha/beta signalling" (**I**) were enriched in LRRFIP1 high pancreatic cancer. *NES* normalized enrichment score

GSEA was further used to identify signaling pathways associated with LRRFIP1. Nine pathways, including "Pancreatic cancer", "Signaling by Wnt in cancer", "Hedgehog signaling pathway", "PI3K/AKT/mTOR_VITD3 signaling", "Integrin signaling pathway", "Integrin-mediated cell adhesion signaling", "RhoA pathway", "Toll-like receptor signaling related to MYD88", and "Interferon alpha/beta signalling" were identified enriched in the pancreatic cancer patients with high LRRFIP1 expression (Fig.  5A–I).

### LRRFIP1 predicts immune cell infiltration in pancreatic cancer

**Fig. 6:**
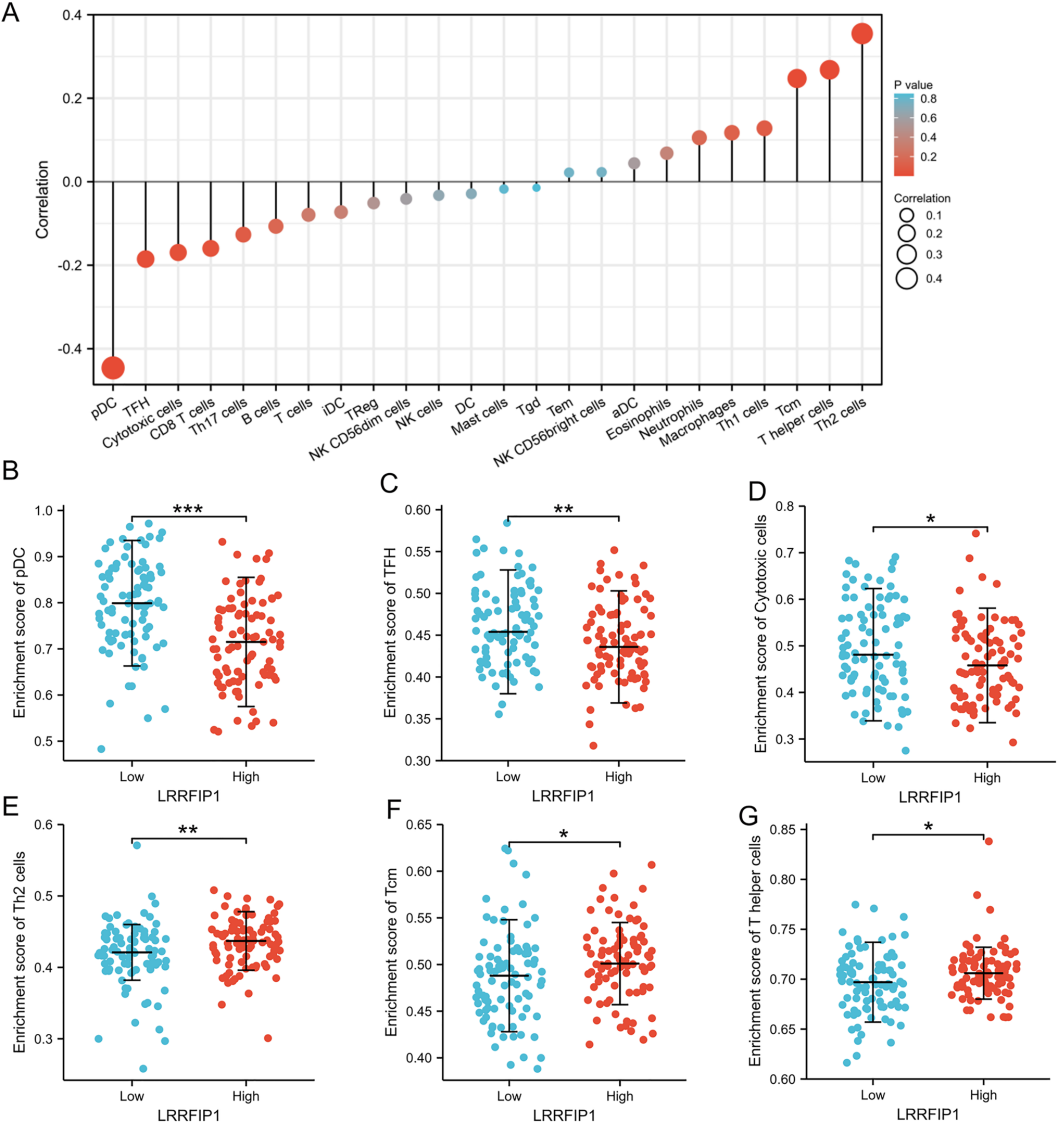
Correlations between LRRFIP1 expression and immune cell infiltration in pancreatic cancer. **A** Correlation between LRRFIP1 expression and 24 types of immune cells in pancreatic cancer. The size of the dots represents the absolute Spearman's correlation coefficient values. **B–G** The expression of LRRFIP1 was related to the enrichment of pDC (**B**), TFH (**C**), Cytotoxic cells (**D**), Th2 cells (**E**), Tcm (**F**), and T helper cells (**G**). DCs, dendritic cells; pDCs, plasmacytoid DCs; aDCs, activated DCs; iDCs, immature DCs; Th, T helper cells; Th1, type 1 Th cells; Th2, type 2 Th cells; Th17, type 17 Th cells; Tcm, T central memory; Tem, T effector memory; TFH, T follicular helper; Tgd, T gamma delta; Treg, regulatory T cells; NK, natural killer. ^*^p < 0.05, ^**^p < 0.01, ^***^p < 0.001 by Student's t-test

Previous studies reported that LRRFIP1 induces the production of type 1 interferon (IFN) and pro-inflammatory cytokines via binding nucleic acids in infectious diseases [22, 23]. Furthermore, LRRFIP1 has recently been identified as the key response prediction gene of PD-1 inhibitors in glioblastoma [24]. Therefore, we analyzed the relationship between LRRFIP1 expression and the immune cells. We applied the R package ssGSEA to investigate the association between the LRRFIP1 expression and 24 different types of immune cells. As shown in Fig. 6A, LRRFIP1 expression was positively correlated with infiltration levels of Th2 cells, T helper cells, Tcm, Th1 cells, macrophages, and neutrophils (*p* < 0.05 for all), whereas it was negatively correlated with infiltration levels of pDC, TFH, cytotoxic cells, CD8 T cells, Th17 cells, B cells, and T cells (*p* < 0.05 for all). Further analysis showed that the infiltration level of pDC was significantly lower in the group of high LRRFIP1 expression than that of low LRRFIP1 expression (*p* < 0.001, Fig. 6B). Similar results were observed in TFH cells (*p* < 0.01, Fig. 6C), and cytotoxic cells (*p* < 0.05, Fig. 6D). Conversely, as shown in Fig. 6E–G, the infiltration levels of Th2 cells, Tcm cells, and T helper cells were significantly higher in the group of high LRRFIP1 expression than that of low LRRFIP1 expression (*p* < 0.05 for all). In short, these results suggest that high LRRFIP1 expression is related to immune cell infiltration in pancreatic cancer.

### Knockdown of LRRFIP1 inhibits proliferation and migration in pancreatic cancer cells.

**Fig. 7:**
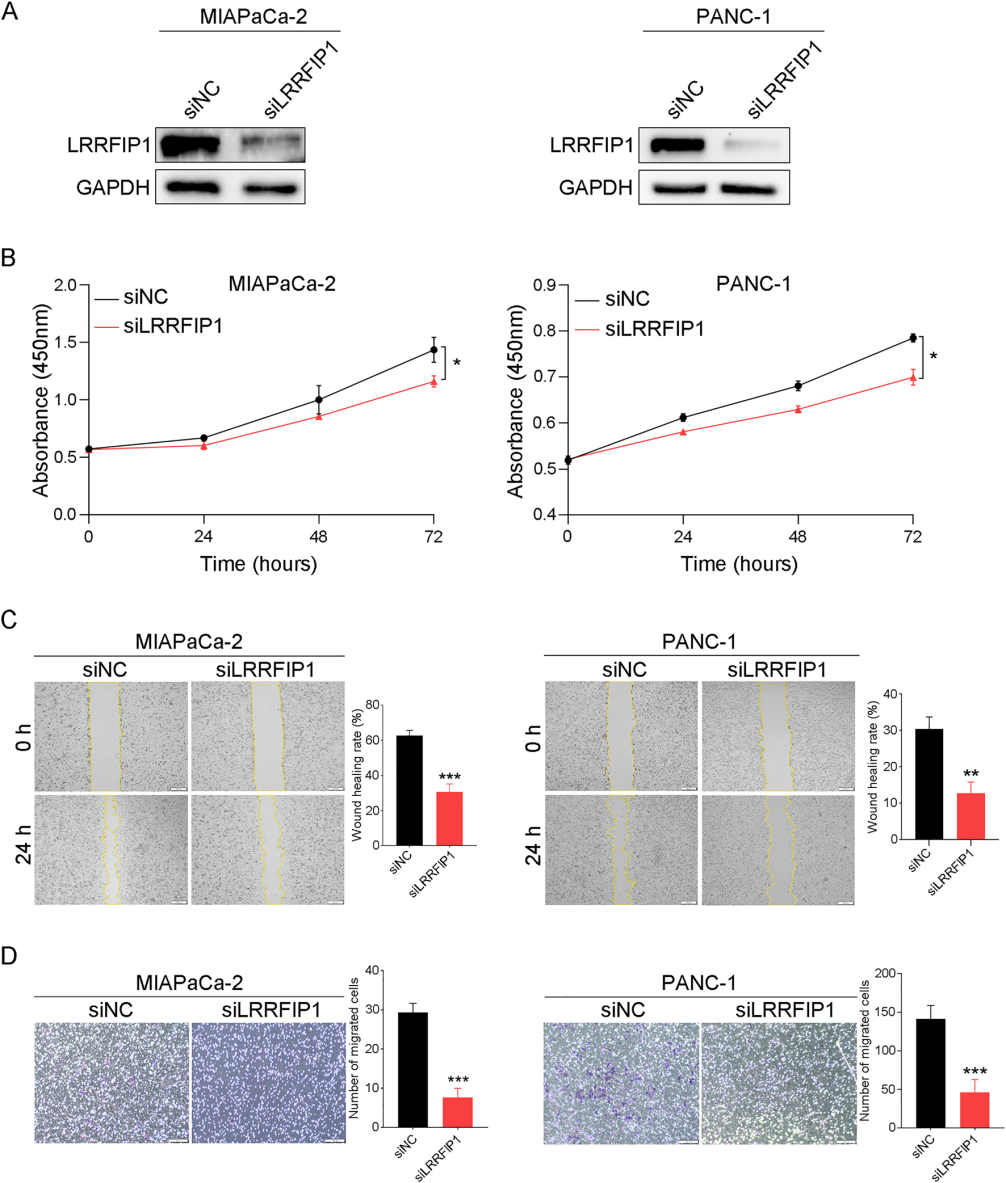
Knockdown of LRRFIP1 inhibits proliferation and migration in pancreatic cancer cells. **A** Western blot analysis of LRRFIP1 expression in MIAPaCa-2 and PANC-1 cells transfected with siLRRFIP1. **B** Cell viability was assayed at 0, 24, 48, and 72 h after knockdown of LRRFIP1. **C** The wound healing rate was significantly decreased in LRRFIP1-silenced cells. Scale bar, 200 μm. **D** Transwell assay showed that LRRFIP1 knockdown reduced the number of migrated cells in MIAPaCa-2 and PANC-1 cells. Scale bar, 100 μm. ^*^p < 0.05, ^**^p < 0.01, ^***^p < 0.001 by Student's t-test

To this end, we set out to investigate the functional role of LRRFIP1 in pancreatic cancer cell models. To evaluate the effects of LRRFIP1 on the growth and metastatic potential of pancreatic cancer cells, we knocked down LRRFIP1 in MIAPaCa-2 and PANC-1 cells (Fig. 7A). The results showed that attenuated LRRFIP1 expression significantly suppressed cell viability (Fig. 7B, *p* < 0.05 for both) and migration ability (Fig. 7C, D, *p *< 0.05 for all). Taken together, these data indicate that LRRFIP1 may play an oncogenic role in tumorigenesis and metastatic potential of pancreatic cancer.

### LRRFIP1 silencing dampens AKT/GSK-3β/β-catenin signaling in pancreatic cancer cells

**Fig. 8:**
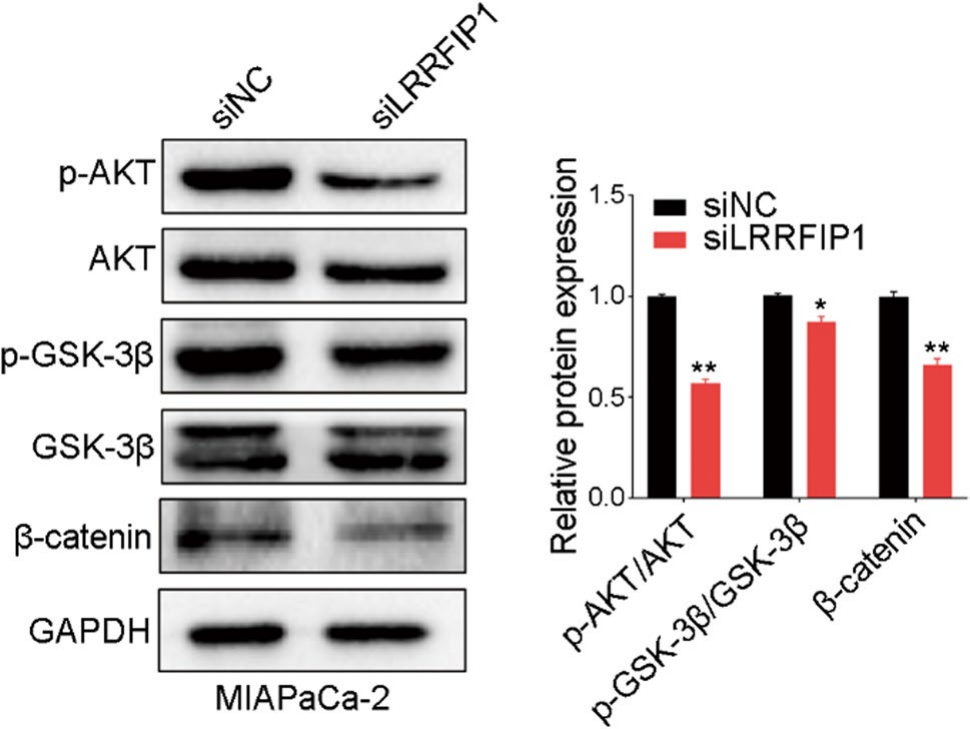
Knockdown of LRRFIP1 inhibits AKT/GSK-3β/β-catenin signaling in pancreatic cancer cells. Western blot analysis of AKT/GSK-3β/β-catenin signaling in MIAPaCa-2 cells with LRRFIP1 knockdown (Left), and the intensities of the indicated proteins were quantified (Right). ^*^p < 0.05, ^**^p < 0.01 by Student's t-test

Given that the above results showed that AKT and Wnt signaling were enriched in pancreatic cancer patients with high LRRFIP1 expression (Fig. 5B, D), and the roles of AKT and Wnt signalings in cancer progression are well established [25, 26]. We measured the protein levels of AKT/GSK-3β/β-catenin signaling in LRRFIP1-silenced cells. Western blotting assay revealed that knockdown of LRRFIP1 resulted in a notable downregulation in p-AKT, p-GSK-3β, and β-catenin in MIAPaCa-2 cells (Fig. 8). Taken together, the findings suggest that LRRFIP1 may influence cell proliferation and mobility in pancreatic cancer through regulation of AKT/GSK-3β/β-catenin signaling pathway.

## Discussion

In the present study, we revealed that LRRFIP1 was upregulated in pancreatic cancer, and its upregulation was associated with poor overall survival, which indicated that LRRFIP1 could act as a prognosis predictor for pancreatic cancer. Moreover, LRRFIP1 was revealed to be associated with the infiltration of multiple immune cell types. Based on the findings that LRRFIP1 might get involved in tumorigenesis revealed by GSEA analysis, subsequent in vitro experiments were conducted, which demonstrated that LRRFIP1 was involved in the progression of pancreatic cancer. Our findings revealed that LRRFIP1 could promote pancreatic cancer progression through the AKT/GSK-3β/β-catenin signaling axis.

LRRFIP1 is abnormally expressed in a variety of cancers [4–7] and regulates a wide range of biological systems and processes, including immune response to microorganisms and autoimmunity, remodeling of cytoskeletal system, signal transduction pathways, and transcriptional regulations of genes [8–11]. In the case of pancreatic cancer, silencing of LRRFIP1 can reverse the EMT of pancreatic cancer cells and increase the gemcitabine sensitivity in pancreatic cancer cells [12, 13]. However, its clinical value for pancreatic cancer diagnosis and prognosis has yet to be determined. Here, we found that LRRFIP1 was significantly upregulated in pancreatic cancer and it could distinguish pancreatic cancer tissues from normal tissues according to ROC analysis. Further research should be performed to confirm its clinical significance for pancreatic cancer diagnosis.

In terms of prognosis, upregulation of LRRFIP1 has been associated with poor overall survival in gliomas [27] and hepatocellular carcinoma [28]. In this study, Kaplan–Meier survival analysis showed that the pancreatic cancer patients with high LRRFIP1 expression had a shorter overall survival than those patients with low LRRFIP1 expression. Similarly, several clinicopathologic subgroups with high LRRFIP1 expression showed lower overall survival than those with low LRRFIP1 expression. Collectively, the above results suggest that LRRFIP1 is an important prognosis biomarker for pancreatic cancer.

Previous studies have reported that LRRFIP1 acts as a mediator in multiple signaling transduction pathways [4]. To explore the functions of LRRFIP1 in pancreatic cancer, we identified the top 210 genes that most positively correlated with LRRFIP1, and these genes were subjected to GO and KEGG enrichment analysis using the "clusterProfile" R package. GO and KEGG enrichment analysis suggests that the LRRFIP1 was involved with "serine hydrolase activity", "serine-type peptidase activity", "serine-type endopeptidase activity", "pancreatic secretion", "protein digestion and absorption", and "fat digestion in pancreatic cancer". The role of LRRFIP1 in these biological terms requires more clarification. We also found ten possible interacting proteins of LRRFIP1 based on PPI networks, including DHX9, DHX36, and MYD88. Furthermore, qRT-PCR demonstrated that knocking down LRRFIP1 decreased the expression of the majority of the interacting molecules. More research is needed to determine how LRRFIP1 affects the expression of these molecules, as well as whether these molecules mediate LRRFIP1's effects on pancreatic cancer.

Immune cell infiltration is one of the hallmarks of solid tumors and plays a critical role in disease progression [29]. Because LRRFIP1 has been linked to immune responses in infectious diseases and glioblastoma [22–24], we proposed the hypothesis that it may play a role in pancreatic cancer tumor immunity. The results of the correlation analysis revealed that high LRRFIP1 expression was negatively correlated with infiltration levels of pDC, TFH, and Cytotoxic cells, while it was positively related to the infiltration levels of Tcm, T helper cells, and Th2 cells; however, the difference in many immune cell populations between tumors with low and high LRRFIP1 expression is modest. It is worth investigating whether it is functionally relevant to pancreatic cancer's malignant behaviors. It is reported that LRRFIP1 can interact with MYD88 [30], a key regulator of DC and Th2 cells [31], and trigger TLR-mediated NF-κB activity in macrophage and primary human PBMCs [30]. However, we found that in tumors with high LRRFIP1 expression, pDC cell infiltration decreased while Th2 cell infiltration increased. We currently do not understand the reasons for this association in pancreatic cancer. Thus, more research is needed to identify whether LRRFIP1 has a regulatory role in the modulation of MyD88 signaling in DC and Th2 cells.

Finally, the results of GSEA identified that nine pathways were associated with LRRFIP1 in pancreatic cancer, including "pancreatic cancer signaling", "Wnt signaling", Hedgehog signaling pathway", "PI3K/AKT/mTOR_VITD3 signaling", "Integrin signaling pathway, "Integrin-mediated cell adhesion signaling", "RhoA pathway", "Toll-like receptor signaling related to MYD88", and "Interferon alpha/beta signaling". The functions of LRRFIP1 in some of these signaling have been proved in recent studies [9–13]. Therefore, it is a reasonable prospect that LRRFIP1 plays multiple roles in the biological process of pancreatic cancer. Here, we found that knockdown of LRRFIP1 in pancreatic cancer cells downregulated the protein levels of p-AKT, p-GSK-3β, and β-catenin, which suggests that knockdown of LRRFIP1 inhibited the proliferation and migration via dampening the activation of AKT/GSK-3β/β-catenin signaling in pancreatic cancer cells. In support, LRRFIP1 was reported to promote cell invasion and migration through the PI3K/AKT pathway in vascular smooth muscle cells [32]. More research is needed to determine whether LRRFIP1 has a regulatory role in other GSEA-identified pathways. It is worth mentioning that LRRFIP1 knockdown had a greater inhibitory effect on cell mobility than on cell proliferation, with cell proliferation being moderately suppressed, indicating that LRRFIP1 plays a dominating role in cell mobility rather than cell proliferation in pancreatic cancer.

We have to point out there are some limitations in this study. The results and implications regarding LRRFIP1 expression and prognostic value in pancreatic cancer were mainly based on an analysis of online public databases and a small cohort of pancreatic cancer patients; therefore, additional research involving larger independent cohorts should be conducted to validate these findings and implications. Moreover, more in vivo and in vitro experiments are required to clarify the role of LRRFIP1 in pancreatic cancer, especially immune infiltration, and to explore the underlying mechanisms.

## Conclusion

In conclusion, our study found that LRRFIP1 was highly expressed in pancreatic cancer, that high LRRFIP1 expression was associated with poor overall survival and that LRRFIP1 was correlated with immune cell infiltration in pancreatic cancer. LRRFIP1 is essential for pancreatic cancer cell motility and has a minor impact on cell proliferation, which may be mediated by AKT/GSK-3β/β-catenin signaling. Our study provides evidence that LRRFIP1 may play a pivotal role in the progression of pancreatic cancer, and it may be a potential biomarker and therapeutic target for pancreatic cancer.

### Supplementary Information

Additional file1 (DOCX 869 KB)

## Data Availability

The datasets used and/or analyzed during the current study are available from the corresponding author upon reasonable request.

## References

[1] Siegel RL, Miller KD, Jemal A (2020). Cancer statistics, 2020. CA Cancer J Clin.

[2] Morrison AH, Byrne KT, Vonderheide RH (2018). Immunotherapy and prevention of pancreatic cancer. Trends Cancer.

[3] Luo G, Jin K, Deng S, Cheng H, Fan Z, Gong Y, Qian Y, Huang Q, Ni Q, Liu C (2021). Roles of CA19–9 in pancreatic cancer: biomarker, predictor and promoter. Biochim Biophys Acta Rev Cancer.

[4] Takimoto M (2019). Multidisciplinary roles of LRRFIP1/GCF2 in human biological systems and diseases. Cells.

[5] Rikiyama T, Curtis J, Oikawa M, Zimonjic DB, Popescu N, Murphy BA, Wilson MA, Johnson AC (2003). GCF2: expression and molecular analysis of repression. Biochim Biophys Acta.

[6] Kostianets O, Antoniuk S, Filonenko V, Kiyamova R (2012). Immunohistochemical analysis of medullary breast carcinoma autoantigens in different histological types of breast carcinomas. Diagn Pathol.

[7] Li JP, Cao NX, Jiang RT, He SJ, Huang TM, Wu B, Chen DF, Ma P, Chen L, Zhou SF (2014). Knockdown of LRRFIP1/ GCF2 by RNAi causes cell growth inhibition and increased apoptosis in human hepatoma HepG2 cells. Asian Pac J Cancer Prev.

[8] Cohen G, Ettinger K, Lecht S, Lelkes PI, Lazarovici P (2014). Transcriptional down-regulation of epidermal growth factor (EGF) receptors by nerve growth factor (NGF) in PC12 cells. J Mol Neurosci.

[9] Ohtsuka H, Oikawa M, Ariake K, Rikiyama T, Motoi F, Katayose Y, Unno M, Johnson AC (2011). GC-binding factor 2 interacts with dishevelled and regulates Wnt signaling pathways in human carcinoma cell lines. Int J Cancer.

[10] Ariake K, Ohtsuka H, Motoi F, Douchi D, Oikawa M, Rikiyama T, Fukase K, Katayose Y, Egawa S, Unno M (2012). LRRFIP1/GCF2 promotes colorectal cancer metastasis and liver invasion through integrin dependent RhoA activation. Cancer Lett.

[11] Shen DW, Pouliot LM, Gillet JP, Johnson AC, Hall MD, Gottesman MM (2012). The transcription factor LRRFIP1 is an upstream repressor of the small GTPAse RhoA, regulating membrane protein trafficking, sensitivity to doxorubicin, and resistance to cisplatin. Mol Pharm.

[12] Douchi D, Ohtsuka H, Ariake K, Masuda K, Kawasaki S, Kawaguchi K, Fukase K, Oikawa M, Motoi F, Naitoh T (2015). Silencing of LRRFIP1 reverses the epithelial-mesenchymal transition via inhibition of the Wnt/beta-catenin signaling pathway. Cancer Lett.

[13] Kawasaki S, Ohtsuka H, Sato Y, Douchi D, Sato M, Ariake K, Masuda K, Fukase K, Mizuma M, Nakagawa K (2021). Silencing of LRRFIP1 enhances the sensitivity of gemcitabine in pancreatic cancer cells by activating JNK/c-Jun signaling. Pancreatology.

[14] Yan W, Wu X, Zhou W, Fong MY, Cao M, Liu J, Liu X, Chen CH, Fadare O, Pizzo DP (2018). Cancer-cell-secreted exosomal miR-105 promotes tumour growth through the MYC-dependent metabolic reprogramming of stromal cells. Nat Cell Biol.

[15] Vivian J, Rao AA, Nothaft FA, Ketchum C, Armstrong J, Novak A, Pfeil J, Narkizian J, Deran AD, Musselman-Brown A (2017). Toil enables reproducible, open source, big biomedical data analyses. Nat Biotechnol.

[16] Clough E, Barrett T (2016). The gene expression omnibus database. Methods Mol Biol.

[17] Pei H, Li L, Fridley BL, Jenkins GD, Kalari KR, Lingle W, Petersen G, Lou Z, Wang L (2009). FKBP51 affects cancer cell response to chemotherapy by negatively regulating Akt. Cancer Cell.

[18] Yu G, Wang LG, Han Y, He QY (2012). clusterProfiler: an R package for comparing biological themes among gene clusters. OMICS.

[19] Hänzelmann S, Castelo R, Guinney J (2013). GSVA: gene set variation analysis for microarray and RNA-seq data. BMC Bioinf.

[20] Bindea G, Kirilovsky A, Waldner M, Obenauf AC, Angell H, Fredriksen T, Lafontaine L, Berger A, Bruneval P (2013). Spatiotemporal dynamics of intratumoral immune cells reveal the immune landscape in human cancer. Immunity.

[21] Jozefczuk J, Adjaye J (2011). Quantitative real-time PCR-based analysis of gene expression. Methods Enzymol.

[22] Yang P, An H, Liu X, Wen M, Zheng Y, Rui Y, Cao X (2010). The cytosolic nucleic acid sensor LRRFIP1 mediates the production of type I interferon via a beta-catenin-dependent pathway. Nat Immunol.

[23] LiuY ZZ, Zhu B, Hu Z, Zeng P, Wu L (2015). LRRFIP1 inhibits hepatitis C virus replication by inducing type I interferon in hepatocytes. Hepat Mon.

[24] Wong D, Yin Y (2023). Immune micro-environment analysis and establishment of response prediction model for PD-1 blockade immunotherapy in glioblastoma based on transcriptome deconvolution. J Cancer Res Clin Oncol.

[25] Song M, Bode AM, Dong Z, Lee MH (2019). AKT as a therapeutic target for cancer. Cancer Res.

[26] Krishnamurthy N, Kurzrock R (2018). Targeting the Wnt/beta-catenin pathway in cancer: Update on effectors and inhibitors. Cancer Treat Rev.

[27] Ma W, Bao Z, Qian Z, Zhang K, Fan W, Xu J, Ren C, Zhang Y, Jiang T (2022). LRRFIP1, an epigenetically regulated gene, is a prognostic biomarker and predicts malignant phenotypes of glioma. CNS Neurosci Ther.

[28] Li J, Tuo D, Cheng T, Deng Z, Gan J (2024). GCF2 mediates nicotine-induced cancer stemness and progression in hepatocellular carcinoma. Ecotoxicol Environ Saf.

[29] Kalafati L, Kourtzelis I, Schulte-Schrepping J, Li X, Hatzioannou A, Grinenko T, Hagag E, Sinha A, Has C, Dietz S (2020). Innate immune training of granulopoiesis promotes anti-tumor activity. Cell.

[30] Dai P, Jeong SY, Yu Y, Leng T, Wu W, Xie L, Chen X (2009). Modulation of TLR signaling by multiple MyD88-interacting partners including leucine-rich repeat Fli-I-interacting proteins. J Immunol.

[31] Arora M, Poe SL, Oriss TB, Krishnamoorthy N, Yarlagadda M, Wenzel SE, Billiar TR, Ray A, Ray P (2010). TLR4/MyD88-induced CD11b+Gr-1 int F4/80+ non-migratory myeloid cells suppress Th2 effector function in the lung. Mucosal Immunol.

[32] Ma Y, Ren Y, Guan J (2019). Knockdown of GC binding factor 2 by RNA interference inhibits invasion and migration of vascular smooth muscle cells. Mol Med Rep.

