# Supplementary material for: The clinical significance and oncogenic function of LRRFIP1 in pancreatic cancer
Source: Discov Oncol. 2024 Apr 18;15:123. doi: 10.1007/s12672-024-00977-3 (PMC11026317; doi:10.1007/s12672-024-00977-3)
Supplement: Supplementary file 1 — Additional file1 (DOCX 869 KB) [file 12672_2024_977_MOESM1_ESM.docx]

Additional Information


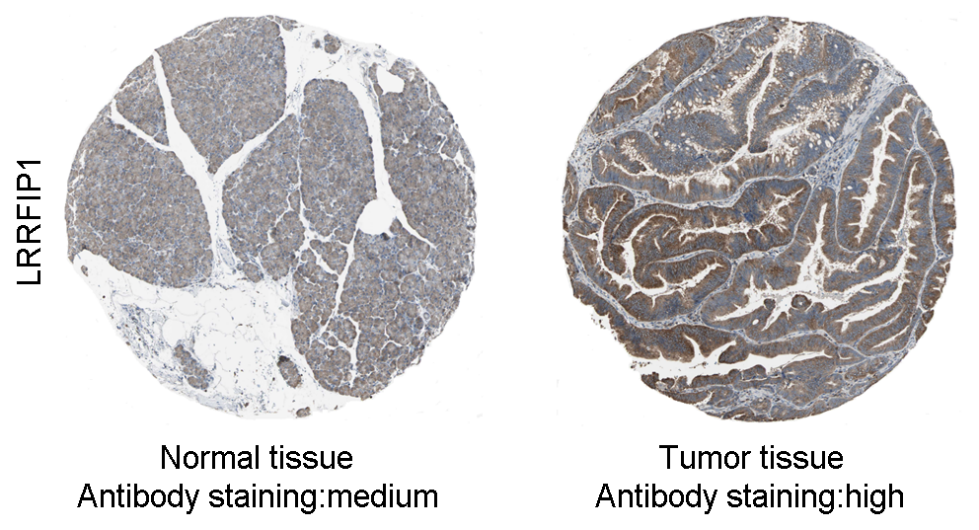


**Fig. S1** Expression of LRRFIP1 in pancreatic cancer in HPA. The immunohistochemical staining of LRRFIP1 in pancreatic cancer tissues and normal tissues was investigated in the HPA database.

**
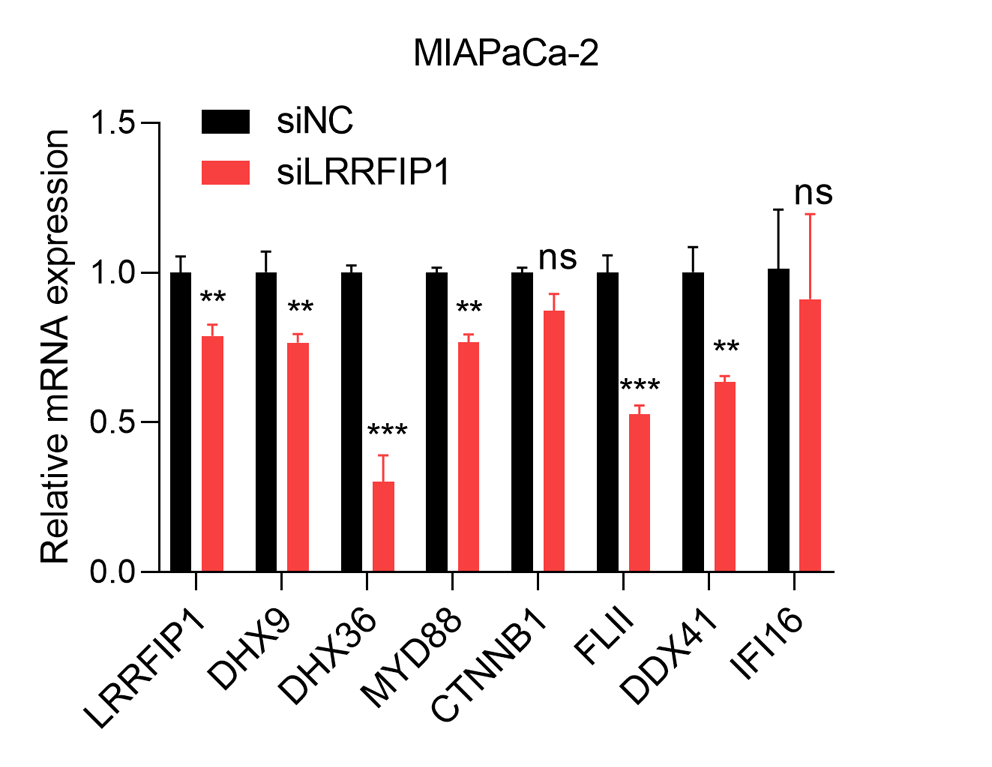
**

**Fig. S2** The effects of LRRFIP1 knockdown on its co-expressed genes. qRT-PCR analysis of LRRFIP1 co-expressed genes in MIAPaCa-2 cells with LRRFIP1 knockdown. ns, not significant. ^**^*p* < 0.01, ^***^*p* < 0.001 by Student's t-test.

**Table S1** PCR primer sequences of target genes

Gene Sequence (5'--3')

(F) GACTTCCGACACCCTCAAT

LRRFIP1

(R) GGGGACCTCTACCATACATT

(F) CGAACCATCTCAGCGACAAAA

DHX9

(R) TGAGGTCCATGCTTATTTGCTC

(F) GGGTCATGGAGGTAACCGAG

DHX36

(R) CTCTCCGCTTCCTTGTTCTTC

(F) GGCTGCTCTCAACATGCGA

MYD88

(R) CTGTGTCCGCACGTTCAAGA

(F) CATCTACACAGTTTGATGCTGCT

CTNNB1

(R) GCAGTTTTGTCAGTTCAGGGA

(F) CTGCAATGACCTGACACGG

FLII

(R) GGTGAGCTGATTTCGGGACAG

(F) GTGCCCTATGTGCCGTTAC

DDX41

(R) GGCTGACGTTGGACTGAGG

(F) AGACTGAAGACTGAACCTGAAGA

IFI16

(R) GAACCCATTGCGGCAAACATA

(F) CAAGGTCATCCATGACAACTTTG

GAPDH

(R) GTCCACCACCCTGTTGCTGTAG

F, forward primer; R, reverse primer.
